# Metal Oxide Gas Sensor Drift Compensation Using a Two-Dimensional Classifier Ensemble

**DOI:** 10.3390/s150510180

**Published:** 2015-04-30

**Authors:** Hang Liu, Renzhi Chu, Zhenan Tang

**Affiliations:** College of Electronic Science and Technology, Dalian University of Technology, No. 2 Linggong Road, Dalian 116023, China; E-Mails: churenzhi123@mail.dlut.edu.cn (R.C.); tangza@dlut.edu.cn (Z.T.)

**Keywords:** sensor drift, metal oxide sensors, classifier ensemble, support vector machines

## Abstract

Sensor drift is the most challenging problem in gas sensing at present. We propose a novel two-dimensional classifier ensemble strategy to solve the gas discrimination problem, regardless of the gas concentration, with high accuracy over extended periods of time. This strategy is appropriate for multi-class classifiers that consist of combinations of pairwise classifiers, such as support vector machines. We compare the performance of the strategy with those of competing methods in an experiment based on a public dataset that was compiled over a period of three years. The experimental results demonstrate that the two-dimensional ensemble outperforms the other methods considered. Furthermore, we propose a pre-aging process inspired by that applied to the sensors to improve the stability of the classifier ensemble. The experimental results demonstrate that the weight of each multi-class classifier model in the ensemble remains fairly static before and after the addition of new classifier models to the ensemble, when a pre-aging procedure is applied.

## 1. Introduction

Metal oxide-type (MOx) sensors are one of several technologies being employed in low-cost air quality monitors. Their size and cost make them ideally suited for portable and remote monitoring [1]. The primary issue in the construction of such systems is the selection and stability of the sensors. A phenomenon known as sensor drift has been recognized as one of the most significant hindrances to the performance of these sensors [2]. To compensate for the drift of gas sensors, all potential avenues, including novel materials, sensor structures and novel algorithms, should be examined.

The algorithms include signal processing and recognition. The main purpose of the signal processing is the separation of drift from real responses, such as orthogonal signal correction (OSC) [3] and common principal component analysis (CPCA) [4]. Additionally, the main purpose of the recognition is to distinguish gases qualitatively or quantitatively without the effect of drift [5,6]. The two types of algorithms are not antagonistic, nor are they alternative, but rather complementary and mutually supportive. This paper focuses on recognition methods for MOx gas sensors. An appropriate pattern recognition method can enhance the stability and accuracy of gas sensors. At one time, analytes were identified using a single classifier model, such as an artificial neural network (ANN), a support vector machine (SVM) or some derivative thereof. Lee *et al.* used a multi-layer neural network with an error back-propagation learning algorithm as a gas pattern recognizer [7]. Polikar *et al.* used a neural network classifier and employed the hill-climb search algorithm to maximize performance [8]. The authors of [9,10] also used an ANN to identify analytes of interest. Xu et al. used a fuzzy ARTMAPclassifier, which is a constructive neural network model that is based on adaptive resonance theory (ART) and fuzzy set theory [11]. Other researchers have used SVMs for classification in e-nose signal processing [5,6,12].

To combine the advantages of different classifiers to improve the accuracy and stability of the analysis, ensemble-based pattern recognition methods have been proposed and have become increasingly important methods used by the chemical sensing research community. Compared with the use of a single classifier model for prediction, classifier ensemble methods have been demonstrated to improve performance, provided that the base models are sufficiently accurate and diverse in their predictions [13,14].

Gao *et al.* used an ensemble of multilayer perceptions (MLPs), which are feedforward artificial neural network models [15], and an ensemble of four base models (namely, MLPs, multivariate logarithmic regression (MVLR), quadratic multivariate logarithmic regression (QMVLR) and an SVM) [16] to simultaneously predict both the classes and concentrations of odors. Shi *et al.* proposed an ensemble of density models, including a k nearest neighbor (k-NN) model, an ANN and an SVM for use in odor discrimination [17]. Vergara *et al.* used an ensemble of multiple SVMs to address the problem of drift in chemical gas sensors [18]. Wang *et al.* also proposed an ensemble of SVMs, similar to that of Vergara *et al.*, except the weight of a classifier is assumed to be reversely proportional to the expected error instead of proportional to the prediction accuracy [19]. Amini *et al.* used an ensemble of SVMs or MLPs on data obtained from a single MOx gas sensor (SP3-AQ2, FIS Inc., Hyogo, Japan) operated at six different rectangular heating voltage pulses (temperature modulation) to identify analytes regardless of their concentration [20]. The experimental results indicated that the accuracies obtained using the ensembles of SVMs or MLPs were nearly equivalent when the same integrating method was used. The weight of each base classifier in the ensembles discussed above is determined prior to the classification phase, and the ensemble process is executed once during this phase. Thus, in the terminology used in this paper, these ensemble methods are said to employ a one-dimensional ensemble strategy. The performance of each of these methods degrades over time due to drift. The performance of future ensemble methods will also inevitably degrade over time if such drift continues to exist. In this paper, we describe a method that can achieve improved performance (or minimize degradation) over time based on a two-dimensional ensemble strategy.

In the remainder of this paper, we first survey existing studies performed by the chemical sensing community concerning problems associated with the use of classifier methods (Section 2). Next, the two-dimensional ensemble strategy proposed in this paper is described, and two pre-aging processes are proposed to improve the stability of the classifier ensemble (Section 3). Finally, the conclusions drawn from the results presented in this paper are discussed (Section 4).

## 2. Related Work

To investigate the drift of gas sensors, the response of the gas sensors in various target gases should be measured over an extensive period of time. For example, Vergara *et al.* [18] used 16 screen-printed MOx gas sensors (TGS2600, TGS2602, TGS2610 and TGS2620, four of each type) that were commercialized and manufactured by Figaro Inc. The resulting dataset comprises 13, 910 recordings of the 16-sensor array upon exposure to six distinct pure gaseous substances, namely ammonia, acetaldehyde, acetone, ethylene, ethanol and toluene. The concentration of each gas ranged from five to 1000 ppmv. The authors mapped the response of the sensor array into a 128-dimensional feature vector, which was generated as a combination of the eight features described in [21] by each of the 16 sensors. The measurements were collected over a 36-month period; for more details about this dataset, refer to [22]. Using this dataset, methods of addressing the drift compensation problem based on signal processing can be examined [23-27].

For the multi-class classification problem, in which each observation is assigned to one of *k* classes, an ensemble method generally combines the decisions of multiple multi-class classifiers that are obtained using various learning algorithms or trained by various datasets to obtain a better predictive performance than could be obtained using any of the constituent classifications individually.

Consider a classification problem in which the set of features **x** serves as the inputs and the class label (a gas/analyte in our problem) *y* serves as the output. At each time step *t*, the batch of examples *S_t_* = (*X_t_*, *Y_t_*) = {(*x*_1_, *y*_1_), …, (*x_m_t__*, *y_m_t__*)} of size *m_t_* is received. The classifier model *f_t_*(*x*) is trained on the dataset *S_t_*. If *S_T_* is the most recent dataset and each *S_t_* (*t* = 1, …,*T*) is a known dataset, then the classifier ensemble *h_T_*(*x*) is a weighted combination of the classifiers trained on each *S_t_*, *i.e.*, $h_{T}\left(x\right)=\sum_{i=1}^{T}\beta_{i}f_{i}\left(x\right)$, where {β_1_, …, β*_T_*} is the set of classifier weights. The ensemble method in its most general form is described in Algorithm 1. The remaining problem concerns how to estimate the weights.

A common and intuitive method is to assign weights to the multi-class classifiers in accordance with their prediction performance on the most recent dataset, namely dataset *S_T_*, as done in [18], because the distributions in the dataset *S_T_* and the test datasets are generally most similar. Other methods of estimating the weights have also been proposed. For example, Wang *et al.* [19] use a weight of β*_i_* = *MSE_r_* − *MSE_i_* for each classifier *f_i_*, where *MSE_i_* is the mean square error of classifier *f_i_* on *S_T_* and *MSE_r_* is the mean square error of a classifier that yields a random prediction.



**Algorithm 1** The classifier ensemble method in its most general form.
**Require:** Datasets *S_t_* = {(*x*_1_, *y*_1_), …, (*x_m_t__*, *y_m_t__*)}, *t* = 1, …, *T*.1:**for**
*t* = 1, …,*T*
**do**2: Train the classifier *f_t_* on *S_t_*;3: Estimate the weight β*_t_* of *f_t_* using dataset *S_T_* using the appropriate technique;4:**end for**5:Normalize the weights {β_1_, …, β*_T_* };**Ensure:** A set of classifiers {*f*_1_, …, *f_T_* } and corresponding weights {β_1_, …, β*_T_* }.


## 3. Two-Dimensional Ensemble Strategy

**Figure 1:**
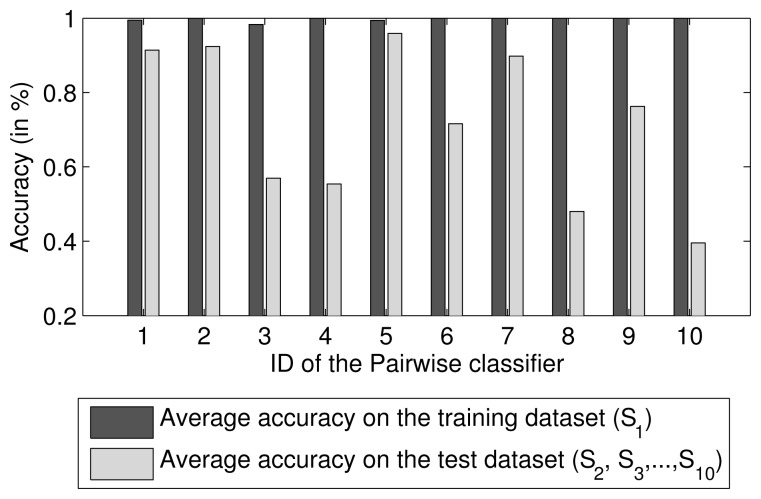
Accuracies of each SVM pairwise classifier trained on the training dataset *S*_1_ and test datasets. *S*_1_ contains feature vectors of five analytes, so 10 pairwise classifiers can be obtained.

Because two-class problems are much easier to solve than multi-class problems, many researchers have proposed the use of a combination of pairwise comparisons for multi-class classification. The combined approach involves voting or probability estimates [28]. Therefore, a multi-class classifier can be regarded as an ensemble of pairwise classifiers. Because the degree of compactness and overlap in each class region of the feature space is different, the separability between each pair of two classes is also different. Thus, the performance of each pairwise classifier differs. For example, for ten SVM pairwise classifiers trained using the first dataset, *S*_1_, collected by Vergara *et al.* [18], their mean predictive accuracies on *S*_1_ and the other datasets are shown in Figure 1. A weighted ensemble of all pairwise classifiers based on their performance should achieve better predictive performance than that of any individual classifier. Thus, a two-dimensional ensemble strategy is proposed in this paper.

### 3.1. Our Approach

In this section, a two-dimensional (2D) ensemble strategy is proposed. This strategy, which is described in Algorithm 2, comprises two ensemble procedures.



**Algorithm 2** Two-dimensional ensemble strategy.
**Require**: Datasets *S_t_* = {(*x*_1_, *y*_1_), …, (*x_m_t__*, *y_m_t__*)}, *t* = 1, …, *T*.1:**for**
*t* = 1, …,*T*
**do**2: Train *k*(*k* − 1)/2 pairwise classifiers on *S_t_*;3: Estimate the first-dimensional weight *w_ij_* for each pairwise classifier using an appropriate technique and normalize the weights;4: Combine the *k*(*k* − 1)/2 binary classifiers to form the multi-class classifier *f_t_*;5: Estimate the second-dimensional weight β_t_ of *f_t_* using an appropriate technique;6:**end for**7:Normalize all second-dimensional weights {β_1_, …, β*_T_* }**Ensure:** A multi-class classifier ensemble, including *T* sets of pairwise classifier models, the weights *w_ij_* for the corresponding multi-class classifiers and the weight β*_t_* for each multi-class classifier.


In the first ensemble procedure, several multi-class classifiers are obtained by combining the weighted pairwise comparisons. First, *k*(*k* − 1)/2 pairwise classifier models are obtained by training a known dataset that contains *k* classes. Given the observation **x** and the class label *y*, we assume that the estimated pairwise class probabilities *r_ij_* of *μ_ij_* = *p*(*y* = *i* | *y* = *i or j*, **x**) are available. Here, the *r_ij_* are obtained from the pairwise classifiers. Then, the goal is to construct an ensemble of the pairwise classifiers to estimate ${\left\{p_{i}\right\}}_{i=1}^{k}$, where *p_i_* = *p*(*y* = *i* | **x**), *i* = 1, …, *k*. The probability estimation method used in this procedure is that proposed by Wu *et al.* [28], because this method is used in LibSVM [29], which has been widely applied. The only difference is that the *r_ij_* used in [28] are replaced with *w_ij_r_ij_*, where the *w_ij_* represent the weights for each pairwise classifier, which are referred to as the first dimensional weights in this paper. They are estimated in accordance with the predictive performance of each pairwise classifier. In this approach, the *w_ij_* represent the prediction accuracy of pairwise comparison on the most recent dataset.

Given *T* known datasets, *T* sets of pairwise classifiers can be obtained. Each set of *k*(*k* − 1)/2 pairwise classifiers can be combined to form a multi-class classifier in the first ensemble procedure. In the second ensemble procedure, these multi-class classifiers are then combined to form a multi-class ensemble using Algorithm 1, where β*_t_* is the prediction accuracy of the *t*-th multi-class classifier on the most recent dataset, which is referred to as the second-dimensional weight in this paper.

Regarding computational complexity, the time needed for calculating classifier models of the proposed approach equals that of other SVM ensembles, such as that proposed in [18,19]. Because a standard multi-class SVM is a combination of pairwise comparisons, in fact a 1D SVM ensemble also need to calculate the same number of binary classifiers as the proposed approach. The time is incremented only in estimating the first-dimensional weight for each pairwise classifier. As this stage is performed offline, training time is not so dramatic a constraint. Moreover, as most parts of the proposed approach can be easily parallelized or distributed, the time can be reduced when dealing with larger problems.

### 3.2. Experimental Section 1

In all of our experiments, we trained multi-class SVMs (one-*vs*.-one strategy) with the RBFkernel using the publicly available LibSVM software. The data used in the experiments was collected by Vergara *et al.* [18]. Because data for toluene are lacking for nearly one year, we used only the data for the remaining five analytes. To avoid over-fitting and to give a reasonable prediction of the performance, the training dataset and test datasets were collected at different periods of time that did not overlap each other, and the test datasets were not used in any way for training the classifiers. The features in the training and test datasets were scaled appropriately to [−1 1]. The kernel bandwidth parameter *γ* and the SVM *C* parameter were chosen using 10-fold cross-validation by performing a grid search in the range [2^−10^, 2^−9^, …, 2^4^, 2^5^] and [2^−5^, 2^−4^, …, 2^9^, 2^10^], respectively, referring to the parameter setting in [18].

The measurements were combined to form 10 datasets, such that the number of measurements was as uniformly distributed as possible. These datasets were numbered in chronological order as *S*_1_, *S*_2_,…,*S*_10_. The larger the index of the dataset, the later it was collected. Note that 5 months separate the collection of *S*_9_ and *S*_10_. This gap is extremely significant for this study for two reasons: (1) it enables us to validate our suggested method using an annotated set of measurements that were collected after an elapsed time of five months; and (2) during this period of time, the sensors were exposed to severe contamination, because external interferents could easily and irreversibly become attached to the sensing layer, because the temperature of the sensors was below the operating temperature.

In the experiments, for a given *T*, we considered that either the values of (*X_t_*, *Y_t_*) in *S_t_* were both known (if *t* ≤ *T*) or *X_t_* was known and *Y_t_* was unknown (if *t* > *T*). Using this data partitioning, we attempted to reproduce real working conditions, in which the system is trained during a limited period of time and is then expected to operate for an extended period of time.

**Figure 2:**
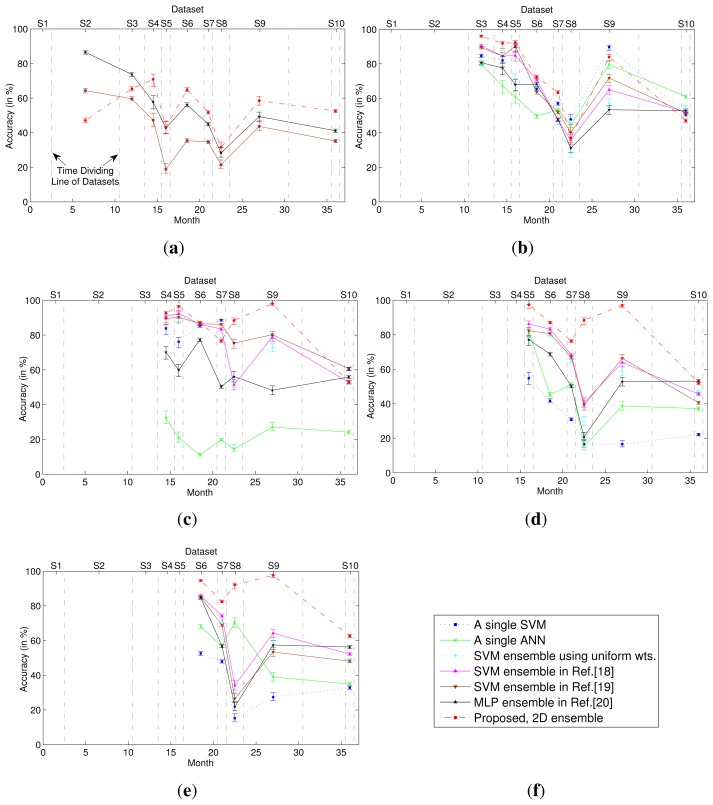
Accuracies and the 95% confidence intervals of the classifiers, including two single-model classifiers, four 1D ensembles and the proposed 2D ensemble. The single-model classifier (SVM or ANN) is trained using *S_T_*. The sub-classifiers in the 1D or 2D ensemble are trained using *S*_1_, *S*_2_,…,*S_T_*, and all of the weights are estimated using *S_T_*. The time range of each dataset is shown, and the vertical dot dash lines represent the time dividing the lines of the datasets. (**a**) The first dataset is known (*T* = 1); (**b**) the first 2 datasets are known (*T* = 2); (**c**) the first 3 datasets are known (*T* = 3); (**d**) the first 4 datasets are known (*T* = 4); (**e**) the first 5 datasets are known (*T* = 5); (**f**) Legend.

The classification performance of the 2D classifier ensemble was compared with those obtained using different classification methods, including single-model classifiers and 1D classifier ensembles. Figure 2 presents the classification accuracies obtained when *T* = 1, 2, 3, 4 and 5, where the first Tdatasets were used for training and subsequent datasets were used for testing. For the 1D and 2D classifier ensembles, all weights were estimated on dataset *S_T_*. For the single-model classifiers (using a single SVM or ANN), the model was trained on dataset *S_T_*. As shown in Figure 2, the 2D classifier ensemble outperformed these single-model classifiers and 1D classifier ensembles when tested on the majority of the unknown datasets, yielding significant improvements in accuracy. Moreover, the larger the number of training datasets, the greater were the advantages of the 2D classifier ensemble.

**Figure 3:**
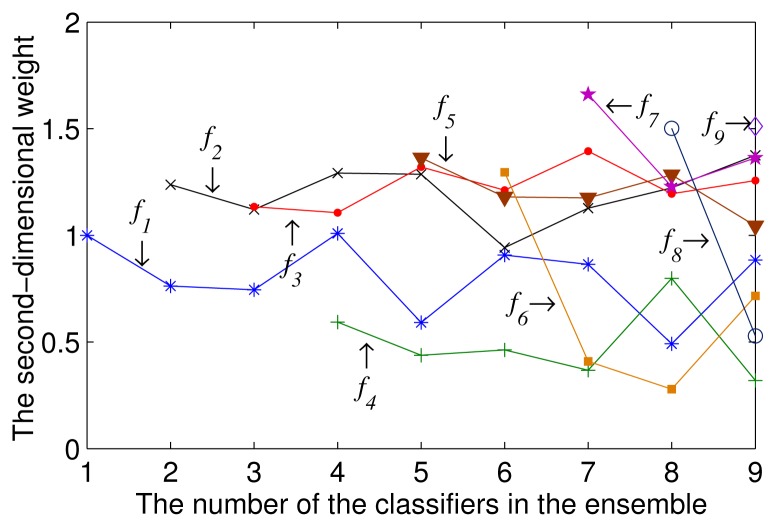
The second-dimensional weights of each multi-class classifier in the ensembles combining different numbers of classifiers, where *f_i_* represents the multi-class classifier trained using the dataset *S_i_*.

To compare the change of the second-dimensional weight of each multi-class classifier in the ensembles combining varying numbers of multi-class classifiers, we linearly scale all of the weights of the multi-class classifiers, so that their sum is equal to the number of the multi-class classifiers in the ensemble. Figure 3 illustrates how the second-dimensional weight of each multi-class classifier in the ensemble changed with *T*. As shown in Figure 3, the weight of each classifier fluctuated significantly. This fluctuation caused the ensemble to become excessively sensitive to the training datasets. Moreover, the weights must be re-calculated after the addition of new classifier models to the ensemble.

**Figure 4:**
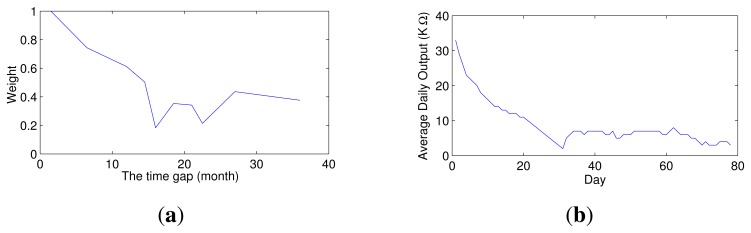
Similarity of computational and physical coefficients. (**a**) The weight of a multi-class classifier in the ensemble for different time gaps between the training dataset and the dataset used to estimate the weight; (**b**) average daily resistance of a gas sensor measured in clean and dry air during the pre-aging process.

The multi-class classifiers in the ensemble are trained using training datasets collected at different times, whereas their second-dimensional weights are all estimated based on the same dataset, namely the most recent dataset (*S_T_*). Thus, the time gap between the training dataset and the dataset that is used to estimate the weight differs for each multi-class classifier. As shown in Figure 4a, this gap significantly affects the weight. If the training dataset of a multi-class classifier is the most recent dataset, then the weight of that classifier is generally the largest among all classifiers in the ensemble; as the gap increases, the weight rapidly decreases. The larger the gap, the higher is the stability of the weight of the multi-class classifier. In other words, the smaller the gap between the training and test datasets, the greater is the accuracy of the multi-class classifier, and as the gap increases, the accuracy initially rapidly decreases and then tends to become stable. This response is similar to that of a gas sensor during the pre-aging process, as illustrated in Figure 4b. A freshly-prepared sensor is highly sensitive, but its sensitivity is unstable. After the pre-aging process, the sensor can maintain a stable performance, but the sensitivity decreases. Thus, gas sensors must be subjected to a pre-aging process to stabilize their performance prior to delivery from the factory. Inspired by the pre-aging process of gas sensors, this paper proposes that the stability of a classifier ensemble can be improved by applying a pre-aging process.

### 3.3. Pre-Aging Procedure

The schedule of traditional ensembles is as following. At first, if given *T* known datasets (*S*_1_, *S*_2_, …, *S_T_*), *T* multi-class classifier models are trained, one on each dataset. All of their weights are estimated using *S_T_*, namely the most recent dataset. Generally, the resulting weight of each model changes greatly after the addition of new models to the ensemble. This section proposes two methods of pre-aging a classifier ensemble to keep the weights of original models stable.

#### 3.3.1. The First Pre-Aging Method

This method is based entirely on the pre-aging procedure for gas sensors. At first, a number of classifier models are trained using sets of data. Then their first- and second-dimensional weights are estimated using a more recent dataset in order to pre-age the ensemble. For example, if given *T* known datasets, the first *T* − 1 datasets (*S*_1_, *S*_2_, …, *S_T_*_−1_) are used to train *T* − 1 separate multi-class classifier models. All of their first- and second-dimensional weights are then estimated using *S_T_*, namely the more recent dataset than the training datasets. In this method, only a portion of the known datasets are used for training, so the multi-class classifier models are fewer than the corresponding models without pre-aging.

#### 3.3.2. The Second Pre-Aging Method

This method is an improvement of the first one. If a number of classification models are trained respectively using some training datasets, their first- and second-dimensional weights are estimated using a combination of these training datasets. For example, if given *T* known datasets, *T* multi-class classifier models are trained, one on each dataset. All of their first- and second-dimensional weights are estimated using a single set combining *S*_1_, *S*_2_, …, *S_T_*. Compared with the first method, this method does not decrease the number of models.

### 3.4. Experimental Section 2

**Figure 5:**
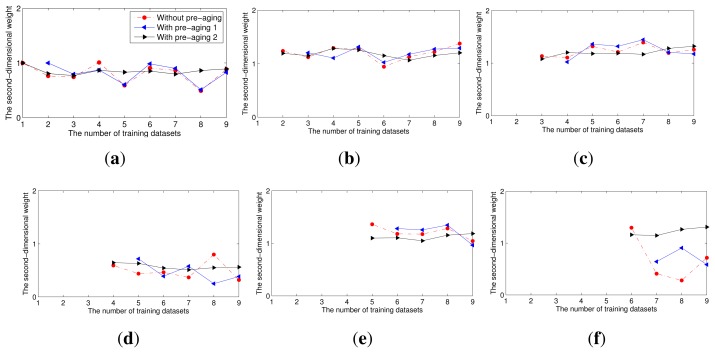
The second-dimensional weights of each multi-class classifier in the ensemble without and with pre-aging when different numbers of known datasets are used for training and pre-aging. (**a**) *f*_1_ trained using *S*_1_; (**b**) the weights of *f*_2_; (**c**) the weights of *f*_3_; (**d**) the weights of *f*_4_; (**e**) the weights of *f*_5_; (**f**) the weights of *f*_6_.

**Figure 6:**
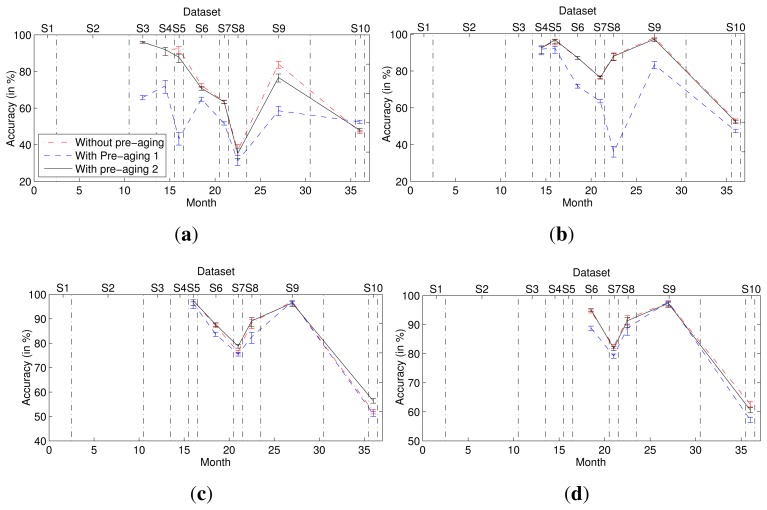
Accuracies and the 95% confidence intervals of the 2D ensembles with and without pre-aging. (**a**) The first dataset is known (*T* = 1); (**b**) the first 2 datasets are known (*T* = 2); (**c**) the first 3 datasets are known (*T* = 3); (**d**) the first 4 datasets are known (*T* = 4).

In this experiment, we compared the second-dimensional weights of each multi-class classifier in the ensemble without and with pre-aging (Figure 5) and the corresponding accuracies (Figure 6). To quantify the change of the second-dimensional weights, we calculated the variance of the second-dimensional weights of each multi-class classifier when the ensemble without or with pre-aging contains different numbers of multi-class classifier models. For example, the variance of *f_i_* is that of the weights of the multi-class classifier trained using dataset *S_i_* when the ensemble contains *i*, *i* + 1,…,9 models separately. As shown in Table 1, for each multi-class classifier model, the variance of its second-dimensional weights in the ensembles with the second pre-aging procedure is least. The results indicate that the second-dimensional weight of each multi-class classifier model in the ensemble with the pre-aging process remains fairly static before and after the addition of new classifier models to the ensemble, especially when the second pre-aging method is used. Moreover, the accuracy of the ensemble with the second pre-aging method is slightly superior to the accuracy of the ensemble without pre-aging.

In the first pre-aging method, only part of the known datasets are used for training, and the most recent dataset is used to estimate weights. Different datasets are used to train the classifier and to estimate its weight, thus preventing the weight of one multi-class classifier from substantially exceeding the weights of the other classifiers. However, given the limited availability of known datasets, the number of multi-class classifiers used in the ensemble must be one fewer than that used in a method with no pre-aging, because the most recent dataset, namely *S_T_*, is not used to train a classifier. Thus, the accuracy of the ensemble obtained using the first pre-aging method is less than those of the other ensembles. The second-dimensional weight of each multi-class classifier increases in stability when the second pre-aging method is used, and the second pre-aging method also eliminates the deficiency in accuracy of the first method. Thus, the ensemble obtained using the second pre-aging method outperforms that obtained using the first pre-aging method.

## 4. Conclusions

We introduced a novel two-dimensional classifier ensemble strategy to solve the gas discrimination problem, regardless of the gas concentration. This strategy is appropriate for multi-class classifiers that consist of combinations of pairwise classifiers, such as support vector machines. We compared the performance of this strategy with those of single-model classifiers and 1D classifier ensembles in experiments based on a public dataset that was compiled over a period of three years. The experimental results demonstrated that over extended periods of time, the 2D ensemble outperformed the other methods that were considered.

The weights of the classifiers were found to fluctuate significantly upon the addition of new models to the ensemble. This fluctuation produces an ensemble that is excessively sensitive to the training datasets. Therefore, we proposed a pre-aging process that was inspired by that applied to the sensors to improve the stability of the classifier ensemble. Two methods of pre-aging were proposed in this paper. We demonstrated that the weight of each multi-class classifier model in the ensemble remains fairly static before and after the addition of new classifier models to the ensemble when a pre-aging procedure is applied, especially using the second proposed method. Moreover, the accuracy of the ensemble obtained using the second pre-aging method is slightly superior to that of the ensemble without pre-aging.

## Figures and Tables

**Table 1:** The variance of the second-dimensional weights of each multi-class classifier in the ensembles without or with pre-aging when different numbers of known datasets are used for training and pre-aging.

**Classifier**	*f*_1_	*f*_2_	*f*_3_	*f*_4_	*f*_5_	*f*_6_
No pre-aging	0.0312	0.0181	0.0104	0.0306	0.0146	0.2048
Pre-aging 1	0.1777	0.0349	0.0298	0.0567	0.0473	0.0390
Pre-aging 2	0.0045	0.0047	0.0062	0.0028	0.0028	0.0063
